# Reactions of Chinese adults to warning labels on cigarette packages: A survey in Jiangsu Province

**DOI:** 10.1186/1471-2458-11-133

**Published:** 2011-02-25

**Authors:** Yu Qin, Ming Wu, Xiaoqun Pan, Quanyong Xiang, Jianping Huang, Zenghui Gu, Zumin Shi, Minghao Zhou

**Affiliations:** 1 Department of Non-communicable Disease Control, Jiangsu Province Centre for Disease Control and Prevention, 172 Jiangsu Road, Nanjing, 210009, PR China; 2 Department of Nutrition and Food Hygiene, Jiangsu Province Centre for Disease Control and Prevention, 172 Jiangsu Road, Nanjing, 210009, PR China; 3 Department of Health Education and Non-communicable Disease Control, Nantong City Centre for Disease Control and Prevention, 189 South-Gongnong Road, Nantong, 226007, PR China; 4 Department of Non-communicable Disease Control, Zhangjiagang City Centre for Disease Control and Prevention, 9 Zhizhong Road, Zhangjiagang, 215600, PR China; 5 Discipline of Medicine, University of Adelaide, 122 Frome Street, Adelaide, SA 5000, Australia

## Abstract

**Background:**

To compare reactions to warning labels presented on cigarette packages with a specific focus on whether the new Chinese warning labels are better than the old labels and international labels.

**Methods:**

Participants aged 18 and over were recruited in two cities of Jiangsu Province in 2008, and 876 face-to-face interviews were completed. Participants were shown six types of warning labels found on cigarette packages. They comprised one old Chinese label, one new label used within the Chinese market, and one Chinese overseas label and three foreign brand labels. Participants were asked about the impact of the warning labels on: their knowledge of harm from smoking, giving cigarettes as a gift, and quitting smoking.

**Results:**

Compared with the old Chinese label, a higher proportion of participants said the new label provided clear information on harm caused by smoking (31.2% vs 18.3%). Participants were less likely to give cigarettes with the new label on the package compared with the old label (25.2% vs 20.8%). These proportions were higher when compared to the international labels. Overall, 26.8% of participants would quit smoking based on information from the old label and 31.5% from the new label. When comparing the Chinese overseas label and other foreign labels to the new Chinese label with regard to providing knowledge of harm warning, impact of quitting smoking and giving cigarettes as a gift, the overseas labels were more effective.

**Conclusion:**

Both the old and the new Chinese warning label are not effective in this target population.

## Background

China is the largest producer and consumer of tobacco in the world. The prevalence of cigarette smoking is above 60% for men aged 15 and above, and 50% of women in this age group and adolescent are passive smokers [1]. Tobacco smoking was estimated as being responsible for approximately 67,300 premature deaths in Chinese adults aged 40 years and over in 2005 [2]. The premature deaths, productivity losses, and a substantial number of preventable diseases and health care costs related to smoking have resulted in a significant economic burden in China [3-7]. However, few people are aware of the harm caused by smoking and passive smoking [8]. Instead, cigarettes are considered a good vehicle for communication, and a popular gift to relatives or friends in China, especially for holidays.

The World Health Organization Framework Convention on Tobacco Control (WHO FCTC), the world's first public health treaty, calls for warning labels to be displayed as large and clear health warnings covering 30% to 50% of the package in the form of pictures, pictograms or text. Every person should be informed of the health consequences, addictive nature, and mortal threat posed by tobacco use and exposure to tobacco smoke. This can be achieved by the warning labels on cigarette packages [9]. In 1991, the Chinese Congress enacted legislation requiring cigarette warnings to state 'smoking is harmful to your health' in Chinese. This warning appeared on one of the side panels of every cigarette package [10]. On 9^th ^of January 2006, WHO FCTC was ratified in China. In 2008, China implemented new regulations according to the FCTC and the legislation. Cigarette warnings were moved from the side panels and covered at least 30% of the front and back of the pack, with Chinese on the front, and English on the back. The warnings include two rotating sets of text in Chinese and English. One set states 'smoking is harmful to your health', and 'quitting smoking reduces health risk'. Another states 'smoking is harmful to your health', and 'quitting smoking early is good for your health' [10].

The purpose of the current study was primarily to compare the difference in reactions to different types of warning labels on cigarette packages with a specific focus on whether the new warning label is better than the previous one and labels used abroad.

## Methods

### Study sites

The study was conducted in 2008 in Nantong and Zhangjiagang cities, Jiangsu Province, one of the economically booming areas in Eastern part of China. Nantong is an urban city, and is a moderate developed city in the Province. Zhangjiagang is a rural area, and belongs to Suzhou City, a highly developed city in the Province.

### Participants

Eligible study participants included in this survey were those aged 18 years and over and those working in hospitals, schools, bus/train stations, government offices, restaurants and bars. Altogether 1000 adults were approached, and 876 participants agreed to participate and finished the survey. All participants were asked to complete a face-to-face interview using a standard questionnaire; informed consent was sought prior to the interview being undertaken. The study was approved by the ethical board of Jiangsu Provincial Centre for Disease Control and Prevention. Verbal consent was obtained from each participant.

### Smoking status and demographic variables

Information was obtained from all participants about their smoking status. Smoking status was measured by asking if participants had ever smoked. Participants were grouped into two categories smokers and former smokers. Smokers were defined as those having smoked at least 100 cigarettes in their lifetime, and those having smoked at least one cigarette per day at the time of the survey. Former smokers were defined as individuals who had quit smoking at least one month prior to the survey and those who smoked at least one cigarette per day prior to quitting. Participants also reported their gender, age, and education level.

### Warning labels of cigarette packages

**Figure 1:**
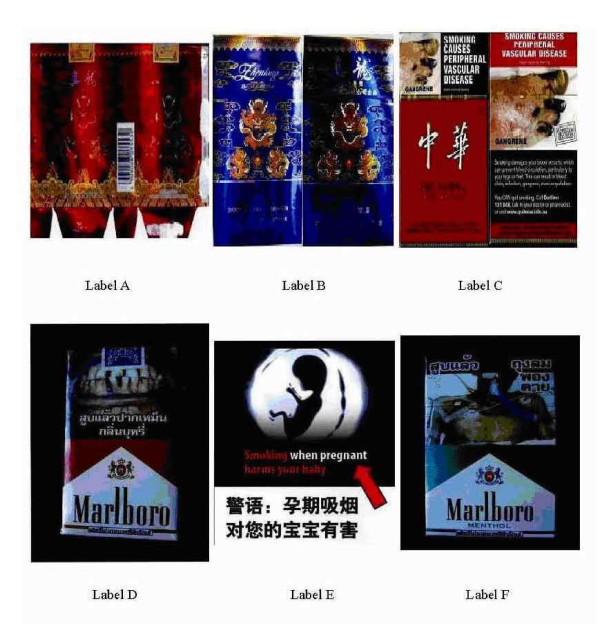
**Six cigarette warning labels**. Label A was the old Chinese warning label, with 'smoking is harmful to your health' in Chinese on one of the side panels of the pack. Label B was the new Chinese warning label, with 'smoking is harmful to your health', and 'quit smoking reduces health risk' on the front and back faces of the package, in Chinese and English respectively. Label C was a famous Chinese brand exported abroad, with text, pictorials, and quitline on the whole front face and 1/3 back face, respectively. The text said 'smoking damages your blood vessels, which can prevent blood circulation, particularly to your legs or feet. This can result in blood clots, infection, gangrene, even amputation.' The other labels were foreign brands. Label D warned that cigarette smoking can result in mouth and oropharynx cancers in text and pictures on 50% of cigarette package. Label E showed smoking when pregnant harms your baby in large area with text and pictures. Label F indicated that smoking can lead to laryngeal cancer in text and pictorial on 50% of cigarette package.

Six warning labels were included in the interview questionnaire (Additional file 1). They were coded A-F. Label A was the old Chinese warning label, with 'smoking is harmful to your health' written in Chinese on one of the side panels of the pack (Figure 1). Label B was the new Chinese warning label, with 'smoking is harmful to your health', and 'quitting smoking reduces health risk' written on the front and back faces of the package, in Chinese and English respectively. Label C was a famous Chinese brand which is exported abroad and includes text, pictorials, and quitline information on the whole front face and 1/3 back face, respectively. The text on the pack reads 'smoking damages your blood vessels, which can prevent blood circulation, particularly to your legs or feet. This can result in blood clots, infection, gangrene, even amputation.' The other labels were foreign brands. Label D warned that cigarette smoking can result in mouth and oropharynx cancers by using text and pictures on 50% of the cigarette package. Label E, also used text and pictures to show that smoking when pregnant harms your baby. Label F indicated that smoking can lead to laryngeal cancer using both text and pictorials on 50% of the cigarette package. All English health warnings were translated into Chinese during the interview.

### The harm warning provided by the label

Making reference to Labels A to F, participants were asked if the each label gave them clear information on the harm which cigarette smoking can have on health and the specific diseases that occur related to cigarette smoking. Participants were also asked if Labels C, D, E, and F gave them clear information on specific diseases smoking can cause (as described above).

### The perceived impact of giving cigarettes as a gift

Three questions on the perceived impact of giving cigarettes as a gift were presented. These included: 1) If you want to use cigarettes as a gift, do the following cigarette labels (A-F) make you change your mind and not do so? 2) If you want to give cigarettes as a gift, which warning label is least likely to stop you using cigarettes as a gift? 3) If you want to give cigarettes as a gift, which warning label is most likely to stop you using cigarettes as a gift?

### The perceived impact on the decision to quit smoking

Participants were asked three questions about the perceived impact of quitting smoking. These include: 1) If you were a cigarette smoker, would the following labels (A-F) make you want to quit smoking? 2) If you were a cigarette smoker, which warning label is most likely to cause you to quit? 3) If you were a cigarette smoker, which warning label is least likely to cause you to quit?

### Knowledge of the FCTC and its provision for cigarette packaging

Participants were asked if they knew that China had ratified the WHO FCTC. If they answered yes, participants were then asked if they were aware of the FCTC provision that health warnings on cigarette packaging should be large, clear, visible and legible.

### Statistic analysis

Univariate and bivariate analyses were conducted to examine how much impact each of the different cigarette warning labels had and the knowledge of the FCTC by age groups, gender, education levels and smoking status. To compare the new Chinese label with international labels, Label C, D, E, and F were aggregated into one group. Chi-square tests were used to assess differences among groups where appropriate. All analyses were conducted using SPSS 13.0 (SPSS Inc., Chicago, IL, USA).

## Results

### General information

Table 1 demonstrates the demographic characteristics of the sample. A total of 876 participants (374 male and 502 female) were involved in the study. The average age was 34.0 ± 11.0 years, a higher proportion of males reported that they were current smokers compared to females and 82.7% of participants had graduated from technical secondary school or higher.

**Table 1:** Characteristics of the study sample

		Male	Female	Total
Number		374	502	876
Age groups (%)	20-29	127(34.0)	211(42.0)	338(38.6)
	30-39	114(30.4)	179(35.7)	293(33.4)
	40 and above	133(35.6)	112(22.3)	245(28.0)
Educational levels (%)	Low (High school and below)	75(20.1)	76(15.2)	151(17.3)
	Medium (technical secondary school)	28(7.5)	105(21.0)	133(15.2)
	High (Junior college and above)	270(72.4)	320(63.9)	590(67.5)
Smoking status (%)	Current smokers	152(40.6)	10(2.0)	162(18.5)
	Former smokers	42(11.2)	4(0.8)	46(5.3)
	Non-smokers	180(48.1)	488(97.2)	668(76.3)

### The harm warning provided by the label

Of the participants, 18.3% said Label A provided adequate information on the harm of cigarette smoking. Among them, 16.5% (14/85) of participants said Label A gave adequate information on the relationship between cigarette smoking and respiratory diseases, including lung cancer, and 16.5% and 3.5% respectively mentioned cancer and cardiovascular diseases. Overall, 31.2% said Label B gave adequate information on the harm of cigarette smoking. Among them, 36.6% said Label B provided adequate information on the relationship between cigarette smoking and respiratory diseases, 5.3% and 3.1% could identify the relationship of smoking with cancer and cardiovascular diseases respectively based on the information on label B. Similar percentage of participants said Label C-F gave adequate information on the harm of cigarette smoking, 90.5% for Label C, 92.7% for Label D, 92.4% for Label E and 92.7% for Label F. Compared to Label A, a higher proportion of participants said that Label B gave them clearer information on the harm of smoking across all subcategories, except those with low education level and current smokers. Labels C-F performed better than Label B in providing harm information for all sub-groups (Table 2).

**Table 2:** The proportion of positive responses to the harm information provided by different cigarette labels by gender, age groups, educational levels and smoking status

		n	Label A	Label B	Label C	Label D	Label E	Label F	**P for Label B vs A**^†^	**P for Label B vs CDEF**^†‡^
Gender	Male	374	18.7	31.0	88.2	89.8	88.8	88.8	< 0.001	< 0.001
	Female	502	17.7	31.3	92.2	94.8	95.0	95.6	< 0.001	< 0.001
Age (yrs)	20-	338	16.3	32.0	90.8	94.1	93.2	93.8	< 0.001	< 0.001
	30-	293	14.0	25.3	92.2	93.2	92.5	92.8	0.001	< 0.001
	40 and above	245	25.7	37.1	88.2	90.2	91.0	91.0	0.006	< 0.001
Education levels*	Low	151	23.8	30.5	89.4	92.1	91.4	94.0	0.196	< 0.001
	Medium	133	15.8	27.1	88.0	90.2	94.0	92.5	0.025	< 0.001
	High	590	17.1	32.4	91.4	93.4	92.2	92.4	< 0.001	< 0.001
Smoking status	Current smoker	162	18.5	23.9	80.9	82.7	83.3	81.5	0.222	< 0.001
	Former smoker	46	17.4	24.1	84.8	89.1	82.6	89.1	0.021	< 0.001
	Non-smoker	668	18.1	33.4	93.3	95.4	95.2	95.7	< 0.001	< 0.001
Total		876	18.3	31.2	90.5	92.7	92.4	92.7	< 0.001	< 0.001

A positive answer means participants can understand the harm information provided by the label.

*low, medium and high education refers to high school and below, technical secondary school, college and above, respectively.

^†^By chi-square test.

^‡^Combination of labels C-F (positive answer to all labels were regarded as positive).

### The perceived impact of giving cigarettes as a gift

Of the participants, 20.8% and 25.2% reported that they would not give cigarettes as a gift to somebody with Labels A and B (respectively) on the package. Over 80% of participants refused to give cigarettes as a gift if the package displayed warning Labels C-F. The proportion of those who would not give cigarettes as gift was higher among female, those who had never smoked and those having a higher educational level. Generally, there was no difference between the sub-groups, in terms of those who would not give cigarettes as a gift, for Label A and Label B, except the proportions were marginally higher among non-smokers and those aged between 30-40 for Label B. When comparing Label B to the combined labels, the proportion of respondents who would not give cigarettes as a gift was higher if any of Labels C-F were on the package (Table 3). The majority of participants (70.4%) considered that Label A was least likely to stop them using cigarettes as a gift, and the proportion was 20.2% for Label B. Almost half of participants (46.8%) considered that Label C was most likely to stop them using cigarettes as a gift, and the proportion was 5.7% and 3.4% for Label A and Label B respectively.

**Table 3:** The perceived impact of not giving cigarette as a gift by gender, age groups, education levels and smoking status

		n	Label A	Label B	Label C	Label D	Label E	Label F	**P for Label B vs A**^†^	**P for Label B vs CDEF**^†‡^
Gender	Male	374	18.4	21.7	80.5	80.5	79.1	81.0	0.273	< 0.001
	Female	502	22.5	27.9	90.2	90.2	89.0	89.6	0.05	< 0.001
Age (yrs)	20-	338	22.5	26.3	87.9	87.9	85.5	87.3	0.244	< 0.001
	30-	293	13.7	19.8	90.1	90.1	89.4	89.8	0.046	< 0.001
	40 and above	245	26.9	30.2	78.8	78.8	78.4	79.6	0.424	< 0.001
Education level	Low	151	23.2	29.8	76.8	76.8	75.5	75.5	0.192	< 0.001
	Medium	133	22.6	24.8	89.5	89.5	85.0	86.5	0.665	< 0.001
	High	590	19.7	24.1	87.6	87.6	87.1	88.5	0.067	< 0.001
Smoking status	Current smoker	162	17.3	21.0	79.6	79.6	75.3	77.8	0.397	< 0.001
	Former smoker	46	22.0	19.6	71.7	71.7	71.7	73.9	0.582	< 0.001
	Non-smoker	668	15.2	26.6	88.6	88.6	88.0	88.8	0.048	< 0.001
Total		876	20.8	25.2	86.1	86.1	84.8	86.0	0.027	< 0.001

*low, medium and high education refers to high school and below, technical secondary school, college and above, respectively.

^†^By chi-square test.

^‡^Combination of labels C-F (positive answer to all labels were regarded as positive).

### The perceived impact on the decision to quit smoking

There were 26.8% and 31.5% of the participants who reported thinking about quitting due to warning Label A and Label B, respectively. In addition, the proportions were all above 80% for Labels C-F. We asked non-smokers if they were smokers, if the labels would impact on a decision to quit smoking. Non-smokers were more likely to quit smoking due to Label C-F, compared to those who were smokers. It was shown that due to the warning on Label B, those more likely to quit were females, those with higher educational level and non-smokers when compared to Label A. Label B was less likely to make the participants quit smoking compared to Labels C-F combined (Table 4). Almost half of participants (43.3%) considered that Label C was most likely to cause them to quit. The proportion was only 4.5% and 3.7% for Label A and B, respectively. The majority of participants (69.9%) considered that Label A was least likely to cause them to quit, and the proportion was 20.2% for Label B. There was no significant difference between smoking status groups in terms of the impact Label A and Label B had on a decision on quit smoking.

**Table 4:** The perceived impact on the decision to quit smoking by gender, age groups education levels and smoking status

		n	Label A	Label B	Label C	Label D	Label E	Label F	**P for Label B vs A**^†^	**P for Label B vs CDEF**^†‡^
Gender	Male	374	27.0	28.6	81.3	80.7	81.0	82.6	0.624	< 0.001
	Female	502	26.7	33.7	91.6	91.6	93.0	93.2	0.016	< 0.001
Age group	20-	338	33.1	33.7	88.8	87.6	89.1	89.1	0.870	< 0.001
	30-	293	20.5	26.6	88.4	87.7	88.4	90.4	0.080	< 0.001
	40 and above	245	25.7	34.3	83.7	85.3	85.7	86.1	0.038	< 0.001
Education level*	Low	151	28.5	34.4	86.1	84.8	84.8	85.4	0.265	< 0.001
	Medium	133	24.8	30.1	88.0	84.2	90.2	91.0	0.336	< 0.001
	High	590	26.8	31.0	87.3	88.1	88.1	89.0	0.108	< 0.001
Smoking status	Current smoker	162	24.7	25.9	76.5	75.9	74.7	79.0	0.798	< 0.001
	Former smoker	46	28.3	26.1	84.8	80.4	80.4	78.3	0.115	< 0.001
	Never smoker	668	13.0	33.2	90.0	90.1	91.6	91.8	0.050	< 0.001
Total		876	26.8	31.5	87.2	87.0	87.9	88.7	0.031	< 0.001

*low, medium and high education refers to high school and below, technical secondary school, college and above, respectively.

^†^By chi-square test.

^‡^Combination of labels C-F (positive answer to all labels were regarded as positive).

### Knowledge of the WHO FCTC and its provision for cigarette packaging

Overall 32.4% of participants knew of the FCTC. Among them, 77.1% and 72.4% were non-smokers and those with the highest educational level, respectively. Furthermore, 75.4% (214/284) knew that China have ratified the FCTC, and 77.5% knew the provision of the FCTC that health warnings on cigarette packaging should be large, clear, visible and legible.

## Discussion and Conclusions

Our study has shown that both the old and new Chinese warning labels have a low effect on the participants' knowledge of the harmful effects of smoking, on giving cigarettes as a gift, and quitting smoking. Labels used abroad were far more effective than the labels used in the Chinese market.

Over 90% of the participants knew 'smoking was harmful to their health', while the knowledge of smoking-related disease, such as cardiovascular diseases, stroke etc. was relatively low. The result is consistent with another report from six cities in China [11]. From our survey, neither the old Chinese label nor the new one is able to provide details of smoking-related disease to smokers or nonsmokers, although there was a difference in the level of information related to the harm of smoking provided by Labels A and B, which may be due to their distinct location on the new pack. The result of no difference in low educational groups between Label A and B suggested that only text warnings cannot provide useful information to poor literacy population. In addition, the text-only labels cannot provide health warnings to current smokers, and smokers were failed to take notice of the difference between the old and new labels, even they take out cigarettes from packages every day.

Our survey showed that text-plus-graphic warning labels were more effective than text-only labels, which is also consistent with other reports [12]. Graphic warnings can clearly express the consequences of smoking, and they are especially useful for populations with poor literacy and difficulty understanding text-based warnings. Moreover, graphic warning labels appear to be an important source of information regarding health risks for non-smokers, which may lead to increased pressure to quit from members of a smokers' social network [13]. More and more countries have mandated the inclusion of graphic imagery on cigarette warning labels (e.g., Australia, Brazil, Canada, Chile, Singapore, Thailand, Uruguay, and Venezuela), with other countries soon to follow (e.g., Belgium and New Zealand) [14].

Warning labels can not only increase awareness of the health hazards, but also provide information on assistance for quitting and can promote interest in quitting. In our study, labels with detailed risk information and graphics had a more effective on the decision to quit. While both the old and the new Chinese labels had less effective with no information on specific smoking-related diseases, and no useful information on cessation. Canadian warning labels on cigarette packs are considered one of the most effective in the world, and are very useful for tobacco cessation. The requirements of the warning label with big, clear and direct health messages provides a strong incentive for smoking cessation [15-17]. Approximately one third of the smokers reported a likelihood of quitting and 20% of smokers reported smoking less, as a result of warning labels with graphic and detailed health risk and cessation information. Smokers were more likely to quit, make an attempt to quit, or reduce their smoking because of increased level of fear and disgust for the labels with text and large graphics [18]. Thus, graphic messages on warning labels appear more effective than text-only messages in promoting quitting [12,14,16,19]. Recent surveys have also shown increased cessation activities due to newly introduced text-and-graphic warnings in countries such as Australia, the United Kingdom, and the United States [19-21].

As a traditional culture, cigarettes are usually considered a valued gift to give, especially on special days, such as Chinese Spring Festival and other holidays. Chinese cigarette packages are always designed with beautiful brand names and graphics, and with one sentence of text warning about the harm but without information related to specific smoking-related diseases. Beautiful designed packs and high prevalence of cigarette smoking in male make cigarettes popular for giving in social communication. Giving cigarette is giving harm. While, the current Chinese warning labels have limited effect on not giving cigarettes as a gift. Compared with foreign warning label requirements, both the old Chinese warning labels and the new ones are relatively weak. The impact will increase if a country changes from smaller to larger and more contrasting warnings [19].

A key limitation of this study concerns the use of a convenience sample which may not be representative of the Jiangsu population. However, purposive selection of groups in six types of work or public places in two cities enabled data collection from a wide range of population segments across a relatively small number of groups [14]. Another limitation was that the educational level of the participants was higher than the general population, thus this is not representative of the average education level of local residents. But, even in the population with higher educational levels, the proportion knowing the harm of smoking and WHO FCTC was not high. We estimate that the proportion is likely to be much lower in the general population. Dissemination of smoking-related knowledge needs be spread widely, especially in smokers and those with lower educational levels.

As the first report in Jiangsu Province, our findings suggest that the new Chinese warning labels are still not effective for this target population. People do not receive sufficient information on the harm of smoking and smoking-related diseases from these labels. In addition, the new warning labels do not effectively increase the desire to quit, or prevent individuals from giving cigarettes to others. The findings from this study indicate that cigarette packaging may benefit from more noticeable, readable, believable and memorable warnings in line with the WHO FCTC and this may be an important policy element in reducing the attractiveness of smoking especially among young adults and teenagers. Warning labels should be part of a larger public health education effort.

## Abbreviations

('WHO FCTC'): World Health Organization Framework Convention on Tobacco Control;

## Competing interests

The authors declare that they have no competing interests.

## Authors' contributions

YQ contributed to the field work, data collection, quality control, analysis, and manuscript writing. MW, QX, and MZ contributed to the implementation in the field and gave advice on the manuscript writing. XP, JH, and ZG contributed to the field work, data collection and quality control. ZS contributed to the statistical advice and critical English review. All authors have read and approved the final manuscript.

## Pre-publication history

The pre-publication history for this paper can be accessed here:

http://www.biomedcentral.com/1471-2458/11/133/prepub

## Supplementary Material

Additional file 1 **Questionnaire for warning labels of cigarette**. As requested by the editor, the questionnaire used in the study was translated into English and presented for the readers. The questionnaire was designed by the China CDC-PUMC-JHSPH Project Group. Any use of it should be noticed to the Group and must be properly cited in any related research products. Click here for file
