# Supplementary material for: Reactions of Chinese adults to warning labels on cigarette packages: A survey in Jiangsu Province
Source: BMC Public Health. 2011 Feb 25;11:133. doi: 10.1186/1471-2458-11-133 (PMC3053246; doi:10.1186/1471-2458-11-133)
Supplement: Additional file 1 — Questionnaire for warning labels of cigarette. As requested by the editor, the questionnaire used in the study was translated into English and presented for the readers. The questionnaire was designed by the China CDC-PUMC-JHSPH Project Group. Any use of it should be noticed to the Group and must be properly cited in any related research products. [file 1471-2458-11-133-S1.DOC]

Additional file

Title: QUESTIONNAIRE FOR WARNING LABELS OF CIGARETTE

City市/县 Zipcode编码_________ Interviewer调查员___________

H．Workplace调查对象 ① hospital医疗机构 ②school学校 ③government offices政府机关 ④public places, including bus/train stations, restaurants and bars公共场所工作人员

H1a. Have you heard about World Health Organization Framework Convention on Tobacco Control ('WHO FCTC')你是否听说过《烟草控制框架公约》？

①yes是 ②no否（go to H2请跳至H2）

H1b. Do you know whether China has ratified the WHO FCTC? 你是否知道我国加入了《烟草控制框架公约》？

①yes是 ②no否 ⑨I don’t know不知道

H1c. Do you know that according to the WHO FCTC the harm warning of cigarette smoking should be large, clear, visible and legible? 你是否知道《烟草控制框架公约》中对烟草制品包装盒的警语的字大而明确、醒目和清晰？

①yes是 ②no否 ⑨I don’t remember记不清

H2（Show labels to interviewee, and ask the following questions请观看图片，然后回答以下问题：）

H2a1. Does label A let you know that cigarettes cause harm to your health? 是否让你知道吸烟对健康的危害？

①if yes, describe specific disease是（具体疾病名称：__________________）

②no否 ⑨I don’t know不知道

H2a2. Does label B let you know that cigarettes cause harm to your health? 是否让你知道吸烟对健康的危害？

①if yes, describe specific disease是（具体疾病名称：__________________）

②no否 ⑨I don’t know不知道

H2a3. Does label C let you know that smoking causes peripheral vascular disease? 是否让你知道吸烟会导致外周血管病变？

①yes是 ②no否 ⑨I don’t know不知道

H2a4. Does label D let you know that smoking causes mouth and oropharynx cancers ? 是否让你知道吸烟会导致口腔癌和喉癌？

①yes是 ②no否 ⑨I don’t know不知道

H2a5. Does label E let you know that smoking causes abortion? 是否让你知道吸烟会导致流产？

①yes是 ②no否 ⑨I don’t know不知道

H2a6. Does label F let you know that smoking causes laryngeal cancer? 是否让你知道吸烟会导致喉癌？

①yes是 ②no否 ⑨I don’t know不知道

| H2b. If you want to use cigarettes as a gift, do the following cigarette labels make you change your mind and not do so? 如果你想送别人卷烟作为礼品，图片中烟盒的包装及健康警示语是否导致你不想再送? | | | | |
| --- | --- | --- | --- | --- |
| label A | ①yes是 | ②no否 | ⑧I don’t know不知道 | ⑨拒绝回答refuse to answer |
| label B | ①yes是 | ②no否 | ⑧I don’t know不知道 | ⑨拒绝回答refuse to answer |
| label C | ①yes是 | ②no否 | ⑧I don’t know不知道 | ⑨拒绝回答refuse to answer |
| label D | ①yes是 | ②no否 | ⑧I don’t know不知道 | ⑨拒绝回答refuse to answer |
| label E | ①yes是 | ②no否 | ⑧I don’t know不知道 | ⑨拒绝回答refuse to answer |
| label F | ①yes是 | ②no否 | ⑧I don’t know不知道 | ⑨拒绝回答refuse to answer |
| H2c. If you want to use cigarettes as a gift, which warning label is least likely to stop you using cigarettes as a gift? 如果你想送别人卷烟作为礼品，哪张图片中烟盒的包装及健康警示语**最不会影响**您送礼？ | | | | |
| ①label A | ②label B | ③label C | ④label D | ⑤label E ⑥label F |
| H2d. If you want to use cigarettes as a gift, which warning label is most likely to stop you using cigarette as a gift?如果你想送别人卷烟作为礼品，哪张图片中烟盒的包装及健康警示语**最能导致你不想再送**？ | | | | |
| ①label A | ②label B | ③label C | ④label D | ⑤label E ⑥label F |
| H2e. If you were a cigarette smoker, would the following labels make you want to quit smoking? 如果你是一个吸烟者，图片中烟盒的包装及健康警示语是否导致你想戒烟？ | | | | |
| label A | ①yes是 | ②no否 | ⑧I don’t know不知道 | ⑨拒绝回答refuse to answer |
| label B | ①yes是 | ②no否 | ⑧I don’t know不知道 | ⑨拒绝回答refuse to answer |
| label C | ①yes是 | ②no否 | ⑧I don’t know不知道 | ⑨拒绝回答refuse to answer |
| label D | ①yes是 | ②no否 | ⑧I don’t know不知道 | ⑨拒绝回答refuse to answer |
| label E | ①yes是 | ②no否 | ⑧I don’t know不知道 | ⑨拒绝回答refuse to answer |
| label F | ①yes是 | ②no否 | ⑧I don’t know不知道 | ⑨拒绝回答refuse to answer |
| H2f. If you were a cigarette smoker, which warning label is most likely to cause you to quit?  如果你是一个吸烟者，哪张图片中烟盒的包装及健康警示语最**能**导致你想戒烟？ | | | | |
| ①label A | ②label B | ③label C | ④label D | ⑤label E ⑥label F |
| H2g. If you were a cigarette smoker, which warning label is least likely to cause you to quit?如果你是一个吸烟者，哪张图片中烟盒的包装及健康警示语最**不能**导致你想戒烟？ | | | | |
| ①label A | ②label B | ③label C | ④label D | ⑤label E ⑥label F |

I1.Do you smoke? 现在你是否吸烟？ ①Yes, daily每天都吸 ②Yes, not daily不是每天都吸 ③quitted戒烟了 ④never smoke从不吸烟

I2.Gender性别 ①male男 ②female女

I3. How old are you你的年龄 ？

I4. Education学历 ①illiterate没上过学 ②primary school小学 ③junior high school初中 ④senior high school高中 ⑤technical secondary school中专/vocational secondary school技校 ⑥university大学/ college and above大专以上
